# Italy’s progress towards the objectives of the national action plan to combat antimicrobial resistance

**DOI:** 10.1371/journal.pone.0347044

**Published:** 2026-04-15

**Authors:** Costanza Vicentini, Stefania di Giacomo, Luca Bresciano, Giulia Fadda, Adriano Grossi, Fortunato D’Ancona, Carla Maria Zotti, SPiNCAR working group

**Affiliations:** 1 Department of Public Health and Paediatrics, University of Turin, Turin, Italy; 2 Department of Infectious Diseases, Epidemiology, Biostatistics and Mathematical Modeling Unit (EPI), Istituto Superiore di Sanità, Rome, Italy; Gabriele d'Annunzio University of Chieti and Pescara: Universita degli Studi Gabriele d'Annunzio Chieti Pescara, ITALY

## Abstract

**Background:**

Antimicrobial resistance (AMR) represents a major public health threat. Italian AMR rates are among the highest in Europe. The Italian National action plan to combat AMR was launched in 2017 and updated in 2022. To monitor its implementation, the self-assessment tool SPiNCAR (Supporto al Piano nazionale per il contrasto all’antimicrobico resistenza) was developed.

**Methods:**

We conducted the first national data collection through SPiNCAR in 2023, assessing the level of implementation across Italian regions and autonomous provinces for the year 2022. Regional SPiNCAR scores were correlated with relevant indicators: healthcare-associated infection (HAI) and antimicrobial use prevalence, as well as antibiotic consumption.

**Results:**

Thirteen regions and autonomous provinces participated. High implementation levels were observed in the SPiNCAR areas of governance, surveillance, HAI prevention and control, and antimicrobial stewardship, while education, stakeholder engagement, and performance assessment showed the lowest scores. Moderate inverse correlations emerged between governance scores and HAI prevalence, and between surveillance/appropriate use scores and antimicrobial use prevalence. A strong inverse correlation was found between performance assessment scores and antimicrobial use prevalence.

**Conclusions:**

This study provides the first national baseline assessment of the level of implementation of the Italian National action plan to combat AMR. Results suggest SPiNCAR could be a useful tool to monitor AMR policies in decentralized health systems. Findings highlight both achievements and gaps; repeated assessments are necessary to guide targeted interventions and reduce regional inequalities.

## Introduction

The growing spread of antibiotic resistance (AMR) is a significant threat for global public health [1]. Among European countries, 75% of infections directly attributable to AMR were estimated to be healthcare-associated [2]. The prevention of healthcare-associated infections (HAIs) is one of the main strategies to tackle AMR and is an achievable goal. Evidence-based infection prevention and control (IPC) interventions could prevent up to 70% of observed HAIs [3]. AMR rates in Italy are among the highest in Europe, and HAIs cause a significant burden [4]. A study based on 2015 data found Italy was one of the European countries with the highest rates of infections due to AMR pathogens. The same study estimated one-third of all deaths attributable to AMR in Europe occurred in Italy [2]. The 2017 ECDC (European Centre for Disease Prevention and Control) *“Country visit to Italy to discuss AMR issues*” highlighted critical issues in management and coordination [5].

The Italian National plan to combat AMR was published in 2017 and updated in 2022 [6]. The plan promotes evidence-based IPC strategies, and underlines the importance of evaluating the impact of interventions on HAI rates. Short-term objectives include the implementation of nationally coordinated and integrated IPC and antimicrobial stewardship strategies, as well as the development of IPC programs that are sustainable and applicable in different health care settings. The plan outlines IPC strategies coordinated at national and regional levels, detailing roles, responsibilities, and required resources [6].

Italy has a universal National healthcare system with a multi-level governance structure. The system aims to provide equal access to standardized levels of health services, regardless of income or geographic location. National health policies and priorities are under the responsibility of the central government (Ministry of health) which determines annual funding and controls the allocation of resources. However, the organization and delivery of health services, including the implementation of the National action plan to combat AMR, is decentralized and entrusted to the country’s 21 regions and autonomous provinces [7]. Regional AMR policies, plans, and programs are enacted, leading to different levels of implementation of the National action plan to combat AMR within the same National healthcare system.

In 2018, following the publication of the first National action plan to combat AMR, the Italian Centre for disease control and prevention (Centro Nazionale per la prevenzione e il controllo delle malattie, CCM, Ministry of health) financed the SPiNCAR (Supporto al Piano **n**azionale per il **c**ontrasto all’**a**ntimicrobico **r**esistenza) project. Within this project, a self-assessment tool was developed, aiming provide a common methodological framework to monitor the level of implementation of the National action plan to combat AMR at regional and local level. The tool promotes continuous improvement by allowing regional governments and trusts to evaluate strengths and weaknesses of their programs against defined standards [8].

This study reports data from the baseline data collection through SPiNCAR, conducted at national level in 2023, with a delay due to the COVID-19 pandemic. The aim of this study was (a) to assess the level of implementation of the National action plan to combat AMR among Italian regions and (b) to validate the SPiNCAR tool through correlation with other relevant indicators.

## Methods

In this study, regional-level responses collected through SPiNCAR were related to data collected through the third ECDC point prevalence survey (PPS) of HAIs and antimicrobial use in acute care hospitals, aggregated at regional level, and antibiotic consumption data retrieved from the Italian Medicine agency (Agenzia Italiana del farmaco, AIFA) national report [9]. Only regions participating in both SPiNCAR and PPS data collections were included in analyses. All sources were linked at the region/autonomous province level. SPiNCAR refers to implementation status as of 31 December 2022; the PPS was conducted between 1 November and 31 December 2022; antimicrobial consumption refers to the 2022 calendar year.

### Data collection – SPiNCAR

SPiNCAR is a self-assessment tool, which monitors through standards and indicators the actions to combat AMR implemented by regions and healthcare facilities, in both human and veterinary fields. For the current study, regional-level responses were considered, which refer to activities carried out by Regional health authorities within their territories. Monitoring through SPiNCAR at national level is coordinated by the Italian National Institute of Health (Istituto Superiore di Sanità, ISS) in collaboration with the Ministry of Health. As per official request by the Ministry of Health, each participating regional healthcare organization designated a single primary contact (regional coordinator) responsible for overseeing data collection. This person could grant data-entry access to additional users to support compilation, but retained final responsibility for validating the dataset and formally closing the submission. A protocol and instructions for data collection were provided, and the ISS assisted regional data collectors throughout the data collection period.

The indicator system was built on the evidence-based guidelines provided by the Italian National action plan to combat AMR, as described by Bravo *et al.* [8]

The self-assessment questionnaire covers seven thematic areas, including: (1) governance, (2) surveillance and monitoring, (3) appropriate antimicrobial use, (4) HAI prevention and control, (5) education and training, (6) alliance among stakeholders, and (7) evaluation of the impact and implementation of the program. Each area consists of two or more standards; each standard includes a number of minimum and additional criteria measuring core and supplementary performance levels respectively. Completing the additional criteria is only possible if all minimum criteria for the relevant standard are met. Criteria are formulated as closed-ended questions (yes/no). Minimum criteria capture foundational structures or processes (e.g., “the Region has formalized the Plan in a specific document”), while additional criteria capture higher-maturity activities (e.g., “the regional Plan defines specific responsibilities and roles for two components of the plan: antimicrobial stewardship, HAIs, veterinary sector”). The full questionnaire is provided as Appendix 1.

Regarding the scoring system, each achieved criterion is assigned a score of one, the sum of scores for all criteria within a standard equals the total score for the standard, and the sum of scores for all standards within an area equals the total score for the area. Percentage scores are computed as the ratio of achieved criteria over total achievable criteria within a standard. No within-standard weighting is applied.

Between June 15, 2023 and September 15, 2023, the first self-assessment was performed by regions concerning the level of implementation as of December 31^st^, 2022. Participation was voluntary, however the budget set aside for the implementation of the 2022–2025 update of the plan will be distributed among regions based on results of this monitoring. Overall, 14 out of 21 Italian regions participated in the survey: Calabria, Emilia Romagna, Friuli-Venezia-Giulia, Lazio, Liguria, Lombardia, Marche, Piemonte, Puglia, Sardegna, Sicilia, province of Trento, Umbria, and Veneto.

### Data collection – PPS

Italy participated in the third ECDC PPS, which was conducted among acute-care hospitals between November 1^st^ and December 31^st^, 2022. Surveillance was coordinated by the University of Turin's Department of Public Health and Pediatrics within a project of the ISS and Italian center for disease control (CCM), Ministry of Health. A web-based software was employed for data collection at national level [10].

Regions were required to participate with a minimum number of acute-care facilities established by the national coordinating team in proportion to: population, number of acute-care hospital bed-days, and number of discharges from acute care facilities. This approach was applied to achieve a national sample which would reflect the regionalized structure of the Italian National health system. Regions were tasked with enrolling facilities within their territories.

The methodology for data collection has been previously described at length [11]. Briefly, an adapted version of the ECDC protocol version 6.1 was applied [12,13]. Data were collected by trained local staff through review of medical records (no direct patient interviews). Data collection was performed on a single day per ward, within three weeks per acute-care facility. All wards within participant hospitals were included (apart from emergency departments); within each ward all patients admitted before 8 a.m. and still present at the time of the PPS were included. Data were collected at hospital, ward, and patient levels. Patient-level variables were collected according to the ECDC PPS protocol; in this ecological analysis we used only aggregated regional indicators (HAI prevalence, antimicrobial-use prevalence, and AMR composite index). Patient-level data included whether the patient was receiving an antimicrobial or had an active HAI on the day of the survey. Microbiological test results were collected for samples isolated from active HAIs, as well as susceptibility testing results for selected AMR markers when available. As per the ECDC PPS protocol, AMR data were collected only for selected combinations of microorganisms and antimicrobial agents. Antimicrobial susceptibility testing data were collected according to the 2019 European committee on antimicrobial susceptibility testing (EUCAST) definitions: susceptible, standard dose; susceptible, increased exposure; resistant. In the Italian PPS, patients positive for healthcare-acquired COVID-19, or admitted for COVID-19, were classified as COVID-19 patients [11].

Overall, 325 hospitals of 19 out of 21 regions participated in the PPS (Abruzzo, province of Bolzano, Calabria, Campania, Emilia Romagna, Friuli-Venezia-Giulia, Lazio, Liguria, Lombardia, Marche, Molise, Piemonte, Puglia, Sardegna, Sicilia, Toscana, province of Trento, Valle d’Aosta, and Veneto), totaling 60,404 patients. For the current study, COVID-19 patients were excluded (n = 1898, 3.14% of included patients).

### Ethics

SPiNCAR monitoring did not involve patient-level data collection; for PPS the written consent of patients was waived, as both SPiNCAR and the national PPS were quality improvement programs coordinated by public entities (ISS, CCM, and Ministry of Health). For PPS, only anonymized data were collected and sent to the national coordinating center. The PPS received the institutional review board approval of the Bioethics Committee of the University of Turin (protocol number 0421518, 29/07/2022).

### Statistical analysis

Data from 13 regions participating in both SPiNCAR and PPS surveillances were included. For regional SPiNCAR results, we only considered the scores related to the minimum criteria, because information on additional criteria was not consistently available—particularly when minimum requirements for a given standard were not fully met. Aggregate analyses were intended to support comparability and monitoring rather than produce summative rankings; therefore, optional criteria were not included in the aggregate score.

Concerning PPS data, HAI and antimicrobial use prevalence were aggregated at regional level as the percentage of patients with at least one active HAI or receiving at least one antimicrobial on the day of the PPS over all included patients in the respective region. HAI prevalence did not include healthcare-acquired COVID-19 cases, as COVID-19 patients were excluded from the current analysis. As defined in the ECDC PPS protocol, the AMR composite index was calculated as the percentage of resistant isolates for ‘first level’ AMR markers over the total number isolates with known antimicrobial susceptibility testing results, aggregated at regional level. The following first-level markers were included:

- *Staphylococcus aureus* resistant to methicillin (MRSA);
- *Enterococcus faecium* and *Enterococcus faecalis* resistant to vancomycin;
- Enterobacterales resistant to third-generation cephalosporins, including *Escherichia coli*, Klebsiella spp., Enterobacter spp., Proteus spp., Citrobacter spp., Serratia spp., and Morganella spp;
- *Pseudomonas aeruginosa* resistant to carbapenems;
- *Acinetobacter baumannii* resistant to carbapenems [12].

Concerning antimicrobial consumption data, we retrieved regional level antimicrobials for systemic use (Anatomical Therapeutic Chemical class J01) consumption for hospital and primary care sectors(from the 2022 Italian Medicine agency report. Hospital consumption was expressed as defined daily dosages (DDDs) per 1000 patients days (pds), whereas community consumption was expressed as DDDs per 1000 inhabitants [9].

Descriptive statistics were used to summarize regional-level SPiNCAR scores per each area and overall, HAI and antimicrobial use prevalences, AMR composite index, and antimicrobial consumption. Quantitative data were summarized using median and interquartile ranges (IQRs) due to non-normal distribution (Shapiro-Wilk tests).

Relationships between considered variables at regional level were assessed using nonparametric Spearman's Rho coefficient, with 95% confidence intervals (CIs) calculated using Fieller, Hartley and Pearson method. Correlation coefficients were interpreted as follows: no correlation Rho 0 ± 0.3; weak correlation Rho ±(0.3–0.5); moderate correlation Rho ±(0.5–0.7); strong correlation ±(0.7–1). Significance was set at two-tailed p < 0.05, with Bonferroni correction for multiple hypothesis testing. All analyses were performed using IBM SPSS (Statistical Package for the Social Sciences), Version 28.0. Armonk, NY: IBM Corp.

## Results

**Fig 1:**
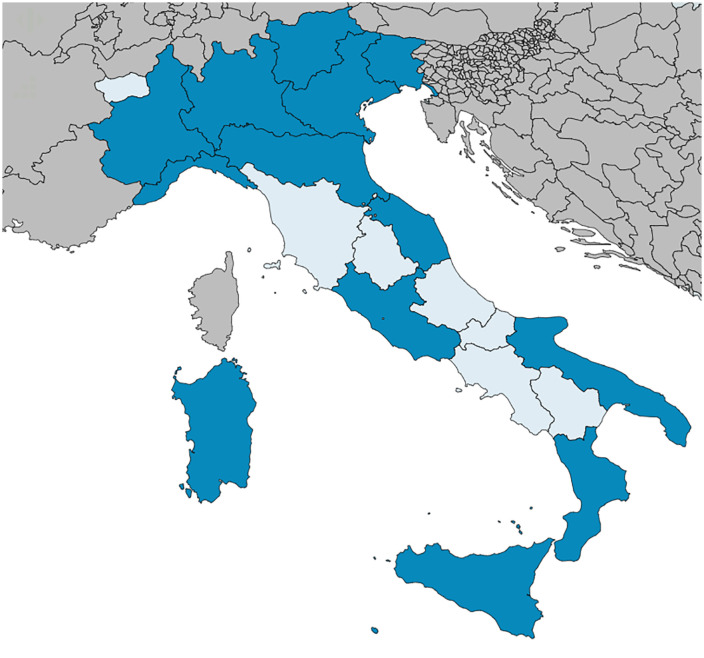
Italian regions included in the study. Regions participating in both SPiNCAR (Support for the National plan to combat antimicrobial resistance) and PPS (point prevalence survey of healthcare associated infections and antibiotic use among European acute-care hospitals), and therefore included in this study, are represented in dark blue. Italian regions not included in the study are represented in light blue. Regions not part of Italy are represented in grey. Created with MapChart.

This analysis included results from 13 regions (Fig 1). Regional-level results per considered variable are summarized in Table 1. Among regions participating in both SPiNCAR and PPS, 48,754 non-COVID-19 patients from 340 acute-care hospitals were included in the PPS. Of these, 4011 had an active HAI on the day of the survey, resulting in an overall HAI prevalence of 8.23 (95% confidence interval, CI 7.98–8.47), with substantial regional variation (median 8.12%, range 3.94–11.46). On the day of the PPS, 21,174 patients received an antimicrobial, resulting in an overall antimicrobial use prevalence of 43.43 (95% CI 42.99–43.87), showing a narrower regional spread (median 43.94%, range 39.61–50.54). Antimicrobial susceptibility testing results were available for 2257 isolates sampled from active HAIs. Among isolates with available antimicrobial susceptibility testing results, 612 were resistant to first-level AMR markers, resulting in an overall AMR composite index of 27.12 (95% CI 25.29–29). The AMR composite index displayed the widest heterogeneity (overall 27.12%, 95% CI 25.29–29.00; median 24.44%, range 10.00–48.61), suggesting marked differences in resistance burden across regions. Across participating regions, implementation was highest for governance and surveillance, while evaluation/performance assessment, education/training, and stakeholder alliance showed the lowest scores (Table 1). Overall SPiNCAR scores ranged from 32 to 82, indicating substantial heterogeneity.

**Table 1:** SPiNCAR scores, healthcare-associated infection (HAI) prevalence, antibiotic use prevalence, antimicrobial resistance (AMR) composite index, and antimicrobial consumption results for 13 regions of Italy, 2022.

SPiNCAR scores	Median score/maximum achievable score (interquartile range, IQR)		Range
Governance	13/17 (15-16)		Apr-17
Surveillance and monitoring	22/29 (17-23)		Sep-29
Appropriate antimicrobial use	8/12 (7-10)		03-Dec
HAI prevention and control	4/4 (2-4)		0-4
Education and training	4/10 (2-7)		01-Oct
Alliance among stakeholders	3/12 (3-4)		01-Aug
Evaluation of the impact and implementation of the program	1/4 (1-4)		0-4
*Overall score*	*56/88 (48-66)*		*32-82*
PPS data	n with condition of interest/total N	Median % (IQR)	Range
HAI prevalence	4011/48,754	8.12 (7.04-8.24)	3.94-11.46
Antimicrobial use prevalence	21,174/48,754	43.94 (42.07-46.46)	39.61-50.54
AMR composite index	612/2257	24.44 (19.31-35.39)	10-48.61
Consumption of systemic antimicrobials (9)	Median (IQR)		Range
Hospital consumption (DDDs/1000 pds)	77.1 (75.5-82.6)		61.1-98.4
Community consumption (DDDs/1000 inhabitants)	7.58 (6.76-10.95)		5.9-18.53

DDDs: defined daily dosages; PPS: point prevalence survey of HAIs and antibiotic use among European acute-care hospitals; pds: patient-days; SPiNCAR: support for the National plan to combat antimicrobial resistance.

**Fig 2:**
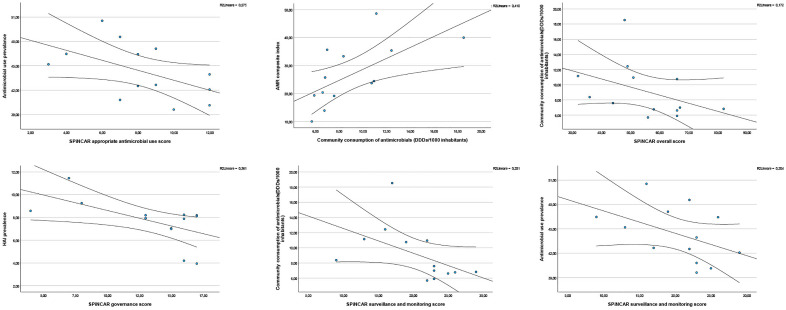
Scatter plots depicting selected significant results of correlation analyses, Italy, 2022. Top row, left to right: SPiNCAR appropriate antimicrobial use score *vs.* antimicrobial use prevalence (p 0.046); AMR composite index *vs.* community consumption of systemic antimicrobials (p 0.003) [9]; SPiNCAR overall score *vs.* community consumption of systemic antimicrobials (p 0.048) [9]. Bottom row, left to right: SPiNCAR governance score *vs.* HAI prevalence (p 0.034); SPiNCAR surveillance and monitoring score *vs.* community consumption of systemic antimicrobials (p 0.012) [9]; SPiNCAR surveillance and monitoring score *vs.* antimicrobial use prevalence (p 0.046). Blue dots represent regional-level results. AMR: antimicrobial resistance; DDDs: defined daily dosages; HAI: healthcare-associated infections; PPS: point prevalence survey of HAIs and antibiotic use among European acute-care hospitals; pds: patient-days; SPiNCAR: support for the National plan to combat antimicrobial resistance.

Results of correlation analyses are summarized in Tables 2–4. Selected significant correlations are depicted in Fig 2.

**Table 2:** Results of correlation analyses, Spearman’s Rho (95% confidence intervals) – SPiNCAR scores *vs.* PPS indicators, Italy, 2022.

SPiNCAR area	PPS data		
	HAI prevalence	Antimicrobial use prevalence	AMR composite index
Governance	−0.59 (−0.87 - −0.4)*	0.07 (−0.51–0.61)	0.01 (−0.65–0.57)
Surveillance and monitoring	0.11 (−0.48–0.64)	−0.56 (−0.85–0)*	−0.54 (−0.85–0.04)
Appropriate antimicrobial use	−0.07 (−0.61–0.51)	−0.56 (−0.85–0.01)*	−0.25 (−0.71–0.37)
HAI prevention and control	0.35 (−0.64–0.47)	−0.13 (−0.64–0.47)	0.23 (−.39–0.7)
Education and training	0.21 (−0.4–0.69)	−0.17 (−0.67–0.43)	−0.24 (−0.71–0.38)
Alliance among stakeholders	0.22 (−0.39–0.7)	−0.12 (−0.64–0.48)	0.18 (−0.43–0.67)
Evaluation of the impact and implementation of the program	0.33 (−0.29–0.75)	−0.77 (−0.93–0.37)*	−0.24 (−0.71–0.37)
Overall score	−0.06 (−0.6–0.52)	−0.38 (−0.78–0.24)	−0.27 (−0.72–0.35)

AMR: antimicrobial resistance; DDDs: defined daily dosages; HAI: healthcare-associated infections; PPS: point prevalence survey of HAIs and antibiotic use among European acute-care hospitals; SPiNCAR: support for the National plan to combat antimicrobial resistance.*uncorrected two-tailed p < 0.05.

**Table 3:** Results of correlation analyses, Spearman’s Rho (95% confidence intervals) – SPiNCAR scores *vs.* antimicrobial consumption data, Italy, 2022.

SPiNCAR area	Consumption of systemic antimicrobials [9]	
	Hospital consumption (DDDs/1000 pds)	Community consumption (DDDs/1000 inhabitants)
Governance	−0.1 (−0.63–0.5)	−0.06 (−0.6 −0.52)
Surveillance and monitoring	0.33 (−0.29–0.75)	−0.67 (−0.9 - −0.18)*
Appropriate antimicrobial use	0.4 (−0.22–0.79)	−0.54 (−0.85–0.03)
HAI prevention and control	0.53 (−0.05–0.84)	−0.28 (−0.73–0.34)
Education and training	0.4 ((−0.21–0.79)	−0.4 (−0.79–0.22)
Alliance among stakeholders	0.51 (−0.08–0.83)	−0.2 (−0.7–0.41)
Evaluation of the impact and implementation of the program	0.21 (−0.4–0.69)	−0.42 (−0.8–0.19)
Overall score	0.51 (−0.07–0.84)	−0.56 (−0.85–0.01)*

DDDs: defined daily dosages; HAI: healthcare-associated infections; pds: patient-days; SPiNCAR: support for the National plan to combat antimicrobial resistance.*uncorrected two-tailed p < 0.05.

**Table 4:** Results of correlation analyses, Spearman’s Rho (95% confidence intervals) – PPS *vs.* antimicrobial consumption data, Italy, 2022.

Consumption of systemic antimicrobials [9]	PPS data		
	HAI prevalence	Antimicrobial use prevalence	AMR composite index
Hospital consumption (DDDs/1000 pds)	0.26 (−0.36–0.72)	−0.25 (−0.71–0.37)	0.24 (−0.37–0.71)
Community consumption (DDDs/1000 inhabitants)	0.01 (−0.56–0.57)	0.51 (−0.08–0.83)	0.75 (0.33–0.92)**

AMR: antimicrobial resistance; DDDs: defined daily dosages; HAI: healthcare-associated infections; PPS: point prevalence survey of HAIs and antibiotic use among European acute-care hospitals; pds: patient-days. **significant result after applying Bonferroni correction.

In uncorrected analyses, higher SPiNCAR governance scores were moderately associated with lower HAI prevalence (Spearman’s ρ = −0.59; p = 0.034). Higher surveillance/monitoring and appropriate antimicrobial use scores were each moderately associated with lower antimicrobial use prevalence (ρ = −0.56; p = 0.046 and ρ = −0.56; p = 0.047, respectively). The strongest association was observed for the SPiNCAR “evaluation of impact and implementation” domain, which showed a strong inverse correlation with antimicrobial use prevalence (ρ = −0.77; p = 0.002). In addition, community antibiotic consumption was inversely correlated with SPiNCAR surveillance/monitoring (ρ = −0.67; p = 0.012) and overall SPiNCAR score (ρ = −0.56; p = 0.048). Finally, the AMR composite index was strongly and positively correlated with community antibiotic consumption (ρ = 0.75; p = 0.003), and this was the only result remaining significant after Bonferroni correction.

## Discussion

This was the first study reporting the level of implementation of the Italian National action plan to combat AMR across regions, assessed through a standardized tool. Monitoring implementation is crucial to integrate and coordinate strategies [8]. From a collaborative perspective, results of monitoring through SPiNCAR could facilitate sharing of best practices: regions with higher implementation levels could become references for those facing greater challenges [14].

Strengths and implementation gaps were assessed at national and regional levels against a national benchmark, providing a roadmap to plan targeted interventions. Considering all participant regions, high levels of implementation were found in the areas of governance, surveillance, HAI prevention and control, and appropriate use of antimicrobials, while the areas with the lowest levels of implementation were performance assessment, education and training, and alliance among stakeholders. Scores for governance and surveillance were consistently high, whereas scores for performance assessment and alliance among stakeholders were consistently low across regions. The highest heterogeneity was found for the areas of HAI prevention and control and education and training.

This assessment highlighted significant room for improvement in multisectoral collaboration across human, animal, and environmental sectors, in a One Health perspective [15]. Awareness and engagement campaigns aimed at diverse stakeholders should be promoted. Successful interventions have been described aiming to promote appropriate antimicrobial use and involving citizens, pharmacists, and general practitioners [16,17]. To translate these priorities into measurable actions, the updated PNCAR 2022–2025 defines specific targets to be achieved by 2025, including at least a 10% reduction in community consumption of systemic antibiotics and at least a 20% reduction in the broad- to narrow-spectrum antibiotic consumption ratio; in pediatrics, it also targets a ≥ 30% increase in the amoxicillin to amoxicillin–clavulanate prescribing ratio. In addition, the plan sets a system-level milestone that by the second half of 2023 all Regions should adopt tools to monitor antimicrobial consumption and appropriateness and progress toward integration and interoperability of information flows to support corrective actions [6].

The second objective of this study was to provide an exploratory validation of SPiNCAR assessment against relevant indicators. Significant correlations were found for several domains. However, most associations were statistically significant only in uncorrected analyses; after Bonferroni adjustment, only the correlation between the AMR composite index and community antibiotic consumption remained significant, and all other correlations should be interpreted as exploratory.

The relation between SPiNCAR governance score and HAI prevalence underscores the importance of institutional commitment and coordination. As in any other field, the availability of a plan does not necessarily ensure that the plan is enacted [18]. Overarching National action plans to combat AMR have been described as ‘plans of plans’: they outline strategic objectives and list recommended actions, combining both new and existing activities enacted by different departments and sectors. For the successful implementation of the plan, clear structures and systems are required to resource and coordinate activities across disciplines [19].

Moderate inverse relations were found between SPiNCAR surveillance and monitoring score and both hospital and community antibiotic use. Surveillance data are essential to inform action at local, regional, and national levels, allowing to enact evidence-based interventions and guide strategy and policy [16]. Further, our study found a strong inverse correlation between SPiNCAR performance assessment and antimicrobial use prevalence, supporting the view of AMR as a dynamic issue which requires regular review and reporting for National action plans to be effective [18,20]. Locally-led follow-up and repeated assessments are essential to prioritize and refine interventions, in particular using standardized tools which allow to demonstrate evidence-based improvements [20].

Concerning antimicrobial stewardship initiatives, SPiNCAR appropriate antimicrobial use score significantly correlated with lower antimicrobial use prevalence in acute-care hospitals. A previous analysis of community antibiotic consumption in Italy found important regional disparities and highlighted the need for interventions to improve prescribing appropriateness targeted towards specific population subgroups [14]. Results of a systematic review of the impact of Japan’s National action plan on antimicrobial prescribing practices also suggest a positive impact in terms of promotion of antimicrobial stewardship activities and reduced antimicrobial consumption [21].

This study had several limitations. First, as a majority but not all Italian regions were included, and as participation was voluntary, we cannot exclude that only those with the highest interest in AMR and implementation levels participated, potentially leading to participation bias. Regions that chose to participate may differ systematically from those that did not. This may affect both the representativeness of national findings and the interpretation of the observed correlations. Responding regions may not reflect the performance or characteristics of non-responding regions, leading to a potential overestimation of the national implementation level. Further, data collection through SPiNCAR is performed as a self-assessment Other limitations pertaining to data collection include possible misunderstandings of the concepts and definitions underlying the SPiNCAR framework, and the fact that filling out additional criteria was only possible if all minimum criteria for the relevant standard were met, leading to a potential underestimation of additional scores. This approach avoids disadvantaging regions that did not assess optional items and limited heterogeneity arising from differing maturity levels across regions. Excluding additional criteria may have underestimated progress in regions implementing advanced practices not captured by minimum criteria. This could slightly compress observed variability at the upper end of performance within some domains.

It must be noted that an ecological, cross-sectional analysis cannot establish causality, and can lead to ecological bias. We used ecological-level Spearman correlations in order to analyse SPiNCAR results and external benchmarks (PPS indicators and antimicrobial consumption) at regional level. Spearman’s rho is appropriate in this context because it is non-parametric, does not assume linearity or normality, and is robust to outliers, which is advantageous with a small sample (n = 13) and potentially skewed indicators. As a construct (convergent) validity check, these correlations provide an interpretable, assumption-light assessment of whether regions with higher SPiNCAR implementation tend to show more favorable external indicators [22]. Considering correlation analyses, assumptions cannot be made on the direction of relations between investigated variables. In particular, based on our analyses we cannot ascertain whether positive results are due to pre-existing levels (and for how long) or to activities outlined by the National action plan. Pre-existing differences in regional healthcare capacity (e.g., staffing levels, availability of dedicated IPC/AMS teams, laboratory and IT resources, and surveillance infrastructure) may systematically influence SPiNCAR performance, with better-resourced regions more able to implement and document required activities—potentially yielding higher scores independent of underlying AMR/HAI burden. Results of our analyses are exploratory; we advise cautious interpretation. The different observation windows may have introduced temporal misalignment across indicators. Further, as the assessment period (PPS in late 2022 and SPiNCAR status at the end of 2022) overlapped with the COVID-19 pandemic, regional IPC capacity and surveillance systems may have been temporarily strengthened or, conversely, strained by staffing shortages and competing priorities. The pandemic may also have influenced antimicrobial prescribing patterns (e.g., changes in empiric use and case-mix) and the implementation and measurement of stewardship activities, potentially affecting SPiNCAR domain scores and contributing to residual confounding in the observed regional differences.

Finally, it was beyond the scope of our analysis to take into account the impact of confounders such as heterogeneity between regions/AP in terms of size, demography, and dedicated economic and human resources. We adopted unweighted regional correlations to preserve equal representation of each unit and to avoid results being driven primarily by a few large regions, which would be expected under population or bed-day weighting. Given this choice, we acknowledge that unweighted analyses may not reflect the national burden; a sensitivity analysis using population (or bed-days) as weights is required to assess the robustness of the main associations. In-depth analyses of political and decision-making processes would also provide a deeper understanding of regional AMR responses.

In conclusion, this study provides preliminary evidence of alignment between SPiNCAR scores and surveillance indicators, suggesting the validity of the SPiNCAR tool to monitor the level of implementation of the Italian National action plan to combat AMR. However, it must be noted that these results do not represent a full validation of the tool.

This study provided a national baseline for future reference and facilitated the comparison of different regional realities. Results are encouraging, but there is still much work to be done. Following the 2022–2025 PNCAR update, SPiNCAR was revised by updating the indicator set to align with the objectives and actions defined in the updated plan. In parallel, data-collection procedures and the web platform were refined to improve standardisation and usability, including: removal of the core/additional criteria distinction, introduction of a “not applicable” response option where relevant, addition of free-text note fields, expanded response formats beyond binary yes/no, and implementation of cascade questions. Criteria were also re-labelled from mandatory/optional to primary/secondary, where primary criteria are explicitly linked to PNCAR 2022–2025 indicators and secondary criteria reflect good practices inherited from the previous SPiNCAR version. Repeated assessments should be conducted to foster continuous improvement, particular focus should be dedicated to reducing regional inequalities and adopting an increasingly integrated approach among human, veterinary, and environmental health. The ongoing commitment of institutions, along with greater collaboration among regions, will be crucial to successfully addressing the global challenge posed by AMR and ensuring the sustainability of the actions taken in the long term.

## Supporting information

S1 File The complete SPiNCAR questionnaire is provided as Appendix 1. (DOCX)
