# Correction: Italy’s progress towards the objectives of the national action plan to combat antimicrobial resistance

**DOI:** 10.1371/journal.pone.0352241

**Published:** 2026-06-22

**Authors:** Costanza Vicentini, Stefania di Giacomo, Luca Bresciano, Giulia Fadda, Adriano Grossi, Fortunato D’Ancona, Carla Maria Zotti, SPiNCAR Working Group

Table 1 was uploaded incorrectly. Please see the correct Table 1 here.

**Table 1:** SPiNCAR scores, healthcare-associated infection (HAI) prevalence, antibiotic use prevalence, antimicrobial resistance (AMR) composite index, and antimicrobial consumption results for 13 regions of Italy, 2022.

SPiNCAR scores	Median score/maximum achievable score (interquartile range, IQR)		Range
Governance	13/17 (15-16)		4-17
Surveillance and monitoring	22/29 (17-23)		9-29
Appropriate antimicrobial use	8/12 (7–10)		3-12
HAI prevention and control	4/4 (2–4)		0-4
Education and training	4/10 (2–7)		1-10
Alliance among stakeholders	3/12 (3–4)		1-8
Evaluation of the impact and implementation of the program	1/4 (1–4)		0-4
*Overall score*	*56/88 (48-66)*		*32-82*
PPS data	n with condition of interest/total N	Median % (IQR)	Range
HAI prevalence	4011/48,754	8.12 (7.04-8.24)	3.94-11.46
Antimicrobial use prevalence	21,174/48,754	43.94 (42.07-46.46)	39.61-50.54
AMR composite index	612/2257	24.44 (19.31-35.39)	10-48.61
Consumption of systemic antimicrobials (9)	Median (IQR)		Range
Hospital consumption (DDDs/1000 pds)	77.1 (75.5-82.6)		61.1-98.4
Community consumption (DDDs/1000 inhabitants)	7.58 (6.76-10.95)		5.9-18.53
